# Prognostic value of p27^Kip1 ^expression in Basaloid Squamous Cell Carcinoma of the larynx

**DOI:** 10.1186/1471-2407-6-146

**Published:** 2006-06-01

**Authors:** Grazia Salerno, Dolores Di Vizio, Stefania Staibano, Giampiero Mottola, Giuseppe Quaremba, Massimo Mascolo, Vieri Galli, Gaetano De Rosa, Luigi Insabato

**Affiliations:** 1 Department of Otolaryngology, University Federico II, Naples, Italy; 2 Department of Biomorphologic and Functional Sciences, Pathology Section, University Federico II, Naples, Italy; 3 Department of Mechanical Engineering for Energetic, University Federico II, Naples, Italy

## Abstract

**Background:**

Very few reports have investigated the role of cell cycle regulators as biomarkers in Basaloid Squamous Cell Carcinoma (BSCC) of the larynx, a definite morphologic, uncommon, very aggressive variant of squamous cell carcinoma. Lower expression of Ki67/Mib-1, a proliferation marker highly expressed in the majority of tumours, and p53, a tumour suppressor protein that can induce an arrest of the G1-S transition, was related to a better prognosis in laryngeal BSCC. In the head and neck, p27^kip1^, a member of the Cip1/Kip1 family of cyclin-dependent kinase inhibitors, has emerged as an independent prognostic factor, able to identify low-expressing tumours with unfavourable course. Up to date the role of this protein was never studied in BSCC. Aim of our study was to investigate the potential prognostic value of p27^kip1 ^levels and their correlation with Ki67/Mib-1 and p53 expression in BSCC of the larynx.

**Methods:**

The retrospective study group consisted of 15 male and 1 female patients, affected by laryngeal BSCC, ranging in age from 44 to 69 years (mean 58). The tumour originated from the supraglottis in thirtheen cases and from the glottis in the remaining three. Ten patients had metastatic cervical lymph nodes at presentation and were classified as N+. Post surgical stage was IV in four patients, III in nine, II in two cases and I in the remaining one. Follow-up ranged from a minimum of 5 months up to 9 years. Paraffin-embedded tissue sections of each laryngeal tumour were analyzed for p27^kip^, Ki67/Mib-1 and p53 expression by immunohistochemistry.

**Results:**

The immunohistochemical study showed p27^kip1 ^expression in 40% of the patients with no evidence of disease (NED) and in none (0%) of the patients dead of disease (DOD), whilst p53 was expressed in 60% of patients in NED status and in 90% of patients in DOD status. Ki67/Mib-1 was positive in 80% of NED patients and in 100% of DOD patients. At multivariate analysis, performed by means of Discriminant analysis, low levels of p27^kip1 ^expression significantly correlated with poor prognosis (P < 0.05).

**Conclusion:**

p27^kip1 ^protein has been shown to be a significant independent prognostic factor in laryngeal SCC. In our series of laryngeal BSCC the resulting data seem to confirm the clinical prognostic relevance of p27^kip1 ^low expression, which directly correlated with biological aggressiveness and consequent shortened survival.

## Background

Basaloid squamous cell carcinoma (BSCC) is a definite morphologic, uncommon, very aggressive variant of squamous cell carcinoma (SCC), which is predominantly localized in the upper aero-digestive tract. In the head and neck region, this distinctive tumour has a strong predilection for extra-laryngeal sites, such as the base of the tongue, the hypopharynx and the supraglottic larynx.

This neoplasm shows a predominant basaloid pattern of growth intimately associated to areas of squamous cell carcinoma and/or adjacent regions of severe dysplasia and carcinoma *in situ*. It mainly affects men in the sixth and seventh decades of life and usually presents at diagnosis in advanced stages, with positive lymph nodes and/or distant metastases.

Since the first cases described by Wain et al [1] in 1986 a few hundreds of cases of BSCC of the aero-digestive tract, head and neck have been reported [2-24]; among them, about 70 cases are from larynx. It is a very rare variant of Squamous Cell Carcinoma (SCC) in this site and to our knowledge, only few isolated cases and very small series were described, with a maximum of 11 reported cases per series. The morphological features are sufficient to diagnose this tumour.

Recently various cell cycle regulators were investigated as biological markers of malignant potential, in an attempt to select which one might influence outcome and the effects of adjuvant therapies. Although conflicting data have emerged regarding the tumorigenesis and the clinical implications of cell cycle regulators in laryngeal SCC, p53 accumulation and Cyclin D1 over-expression have been consistently associated with poor prognosis [25]. The cyclin-dependent kinase inhibitor p27^kip1^is recognized as a negative regulator of the cell-cycle, since it blocks progression from the G1 to the S phase by binding cyclin E-CDK2 and inhibiting their activities. Reduced expression of p27^kip1^is related to progression in precancerous laryngeal lesions [26] and to cell proliferation and poor prognosis in laryngeal squamous cell carcinoma [27-33].

Few reports have investigated the use of cell cycle regulators as prognostic factors in BSCC of the larynx. Ki67/Mib-1 and P53 status were examined in two small series of BSCC of the larynx, resulting in absence of significant correlation of their levels with the clinical findings [34,35]. Recently, Marioni et al [21] investigated the expression of endoglin (CD105), a useful marker for identifying proliferating endothelium involved in tumour angiogenesis, in head and neck BSCCs and SCCs. They did not find any significant differences between the two groups. Rodriguez Tojo et al [22] retrospectively studied the immunoexpression of p53, Ki67/Mib-1 and E-cadherin in 11 cases of laryngeal BSCC, showing that the lower expression of Ki67/Mib-1 and p53 was related to the better prognosis and in all the cases E-cadherin was down regulated.

This study adds 16 new cases of BSCC of the larynx and investigates the expression and the potential prognostic role of cell cycle regulator p27^kip1 ^together with its correlation with Ki67/Mib-1 and p53 expression in BSCC of the larynx.

## Methods

### Selection of cases

From the archival files of the Department of Biomorphologic and Functional Sciences, Pathology Section of the University "Federico II" of Naples, 16 cases of BSCC of the larynx were selected from January 1987 to December 2000 by a review of all laryngo-hypopharyngeal cancers reported. Only tumours showing a prominent basaloid pattern were included in the study. For each case, Hematoxylin-Eosin stain sections confirmed the original diagnosis. All cases fulfilled the basic histological features of BSCC [1].

The clinical records of the patients, i.e., site, TNM status, treatment and follow-up data (length of time since surgery and disease-free interval time to recurrence or death from surgery) are reported in Table 1.

Our series of BSCC consisted of 15 men and 1 woman, whose age at diagnosis ranged from 44 to 69 years (mean age 58). All patients were heavy smokers, with smoking histories ranging from 10 to 30 packets/month. Symptoms at presentation were hoarseness, throat discomfort and dysphagia. Bleeding was an associate symptom in two cases only. The tumour originated from the supraglottis in 13 patients and from the glottis in the remaining three ones; ten patients were N+ at presentation (62.5%) and none of the patients had distant metastasis at the first examination. Post surgical stage was IV in four patients (25%), III in 9 cases (56.2%), II in 2 cases (12.5%) and I in the remaining patient (6.3%). Follow-up ranged from a minimum of 5 months to 9 years.

The primary neoplasm and all regional lymph nodes were surgically excised in 14 cases by either a total laryngectomy or supraglottic laryngectomy, depending on the size and location of the tumour; laser surgery was the preferred surgical approach in the remaining two patients. Eleven patients (68.7%) received postoperative radiotherapy

### Ethical board and informed consent

The Italian law, with reference to the present research, does not provide for the Ethical Committee approval (see *"Circolare del Ministero della Salute" *no. 6 dated September 2^nd ^2002 and DLgs no. 211/2003).

Following the indication of Italian DLgs no. 196/03 (Codex on Privacy) a written consent was obtained from the alive patients.

### Immunohistochemistry

4 μm sections for each case were pre-incubated in a microwave oven for 15 minutes in 10 mmol/L, 6.0 pH buffered citrate followed by the immunohistochemical procedure for Ki67/Mib-1, p53, and p27^kip1^. Ki67/Mib-1 (DAKO) diluted 1:50; p53 (BIOGENEX, San Ramon, CA,) diluted 1:300; p27^kip1 ^monoclonal antibody (Transduction Laboratories, Lexington, KY) diluted 1:500. The conventional avidin-biotin complex procedure was applied according to the manufacturer protocol and the nuclei were counterstained with Mayer's hematoxilin and cover slipped with a synthetic, xylene based, mounting medium. Five different high magnification fields of represented areas of tumours were evaluated.

For each antibody only the neoplastic cells showing a definite nuclear staining were considered as positive and a semi quantitative evaluation was performed. High expression of p27^kip1^, p53 and Ki67/Mib-1 proteins was defined when >50%, >10%, and >10% of cell nuclei were positive, respectively; 0 and 1 were used to discriminate between low and high expressing tumours [36][37]. Two observers, blinded to the follow-up data of single cases, independently evaluated the results. As internal control for p27^kip1 ^immunostaining, resting lymphocytes were used.

### Statistical analysis

Discriminant analysis with a stepwise elimination of variables was performed to build up a predictive model of group membership based on observed characteristics of each case [38]. The model included the following variables as risk factors: age, tumour site, nodal status at presentation, adjuvant radiotherapy, p27^kip1^, p53 and Ki67/Mib-1 expression. In the statistical evaluation, the level of significance used was P < 0.05. The Wilks' lambda method was used for selecting the test set to assess the success of the discriminant function and for choosing the discriminant variables [39].

The Box's Test of Equality of Covariance Matrices was performed to test the assumption that the population under study had a multivariate normal distribution and equal variance-covariance matrices. The amount of explained variance was calculated by co linearity diagnostics and multivariate methods [40]. Finally, the sample was divided in two groups depending on the outcome: patients dead of disease (DOD) and patients with no evidence of disease (NED).

The whole analysis were performed by means of statistical software for Windows (SPSS 10.0, Chicago, III)

### Results

Ten patients (62.5%) died of disease (DOD) despite of salvage surgery and radiotherapy: among them, seven (63.6%) died in the first five years after surgery. Four patients (25%) have no evidence of disease (NED) actually. One patient (6.3%) had a successful treatment for a neck recurrence occurring in the first year after primary surgery and he was disease-free after 9 years. One patient (6.3%) is alive with disease (AWD), i.e. a neck recurrence occurred 4 years after the primary surgery, that was treated with associate radio- and chemotherapy.

**Figure 1:**
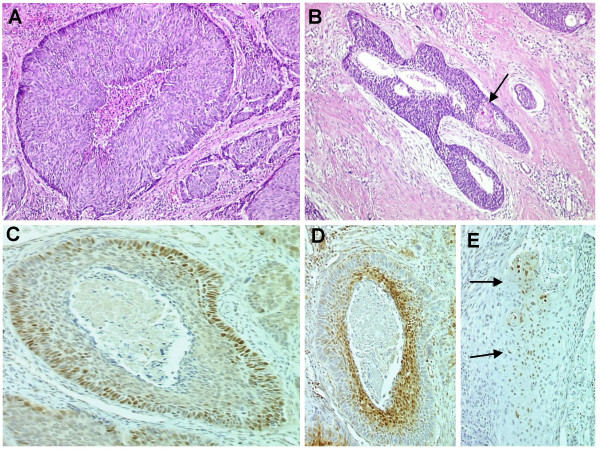
**A** Basaloid nests displaying central necrosis without keratinization. **B** Basaloid cell nests showing an irregular lobular pattern of growth with a focal squamous differentiation (arrow). **C** Immunohistochemical stain with Ki-67/Mib1 showing strongly positive tumour cells at the periphery of the nest. **D** Basaloid nest showing p27^kip1 ^positive staining tumour cells toward the central of the nest, whereas the cells at the invading front of the tumour are negative.  **E** p27kip1 positive staining in the areas of squamous cell differentiation.

Histologically, in nine cases (56.3%) the lesions were composed of basaloid cell nests showing an irregular lobular pattern of growth with a focal squamous differentiation; a small amount of squamous cell carcinoma was focally seen (Fig. 1A–B). The amount of squamous cell carcinoma was more conspicuous in the remaining seven cases (43.7%) that showed a combination of basaloid and squamous areas differently intermixed to each other, the basaloid component being widely prominent. The neoplastic cells had a typical scant cytoplasm, with round to oval, hyperchromatic or, occasionally, large and vesicular nuclei without prominent nucleoli. Mitotic figures and nuclear pleomorphism were frequently observed in all cases. Some basaloid nests displayed central necrosis without keratinization, whereas the cells at the invading front of the tumour tended to show peripheral nuclear palisade arrangements. Areas of hyalinization were present.

There was no variability in the pattern of staining among the samples and the discrepancy between the two observers was negligible. Intratumoural variability was not present. Raw data with median % score and range for each antibody and each case are reported in Table 2.

The immunohistochemical study showed that p27^kip1 ^was highly expressed in 40% of the NED patients and, strikingly, in none (0%) of the DOD patients, whilst p53 and Ki67 were highly expressed respectively in 60% and 80% of the patients in NED status and in 90% and 100% of the patients in DOD status (Table 3). At multivariate analysis high p27^kip1 ^expression demonstrated to be significant (p = 0,01). No significant inverse correlation was found between p27^kip1 ^expression and p53 or to Ki67/Mib-1 expression in DOD and NED patients groups. When the clinical series was globally evaluated, a slight trend for inverse correlation (P = -0,33 for p53 and P = -0.447 for Ki67/Mib-1) resulted.

Interestingly Ki67 staining showed an intense signal in the tumour cells at the periphery of the nests (Fig. 1C), while the internal cell counterpart, facing the degenerating, necrotic centre of the tumour nests, occasionally showed positive tumour cells. In an inverse manner, p27^kip1 ^staining, when present, was specifically polarized, highlighting the cells toward the central necrosis, whereas the basal cells at the invading front of the tumour, showing peripheral nuclear palisading arrangements, were negative (Fig. 1D). p27^kip1 ^positive staining was characteristically revealed mainly in the areas of squamous cell carcinoma (Fig. 1E).

Backward elimination of variables by stepwise procedure (at each step, removing the variable that contributes least to group discrimination) isolated the variable p27^kip1 ^(P = 0.01). The 81,3% of subjects were correctly well classified as belonging to the group membership (Tabs 4, 5).

## Discussion

Although Squamous Cell Carcinoma (SCC) represents the majority of laryngeal cancers, new entities with more severe prognostic implications have been described. One of them is Basaloid Squamous Cell Carcinoma (BSCC), first reported as a clinical-pathologic entity by Wain et al in 1986 [1]. This rare tumour is a histological distinctive variant of SCC with predilection for head and neck region, generally presenting in advanced stage at diagnosis, characterized by aggressive clinical course and extremely poor prognosis.

The survival of patients with head and neck cancer has not changed in the last thirty years [40-42]. The progresses of the classical therapeutic strategies, such as irradiation and surgery, have been unsuccessful in improving the survival rate for these patients. Clinical general status, histopathology examination and tumour classification still remarkably influence the treatment plan.

Several prognostic criteria have been hypothesized to play a role in SCC outcome in the past two decades. Some of the traditional parameters (TNM classification) have been considered useful in predicting the clinical behaviour of this tumour. Nevertheless, the need of finding more reliable predictors of the biological behaviour in single case has become mandatory, with the resultant recent studies on the role of oncogenic markers in tumour onset and progression of SCC [30,44-46]

To the best of our knowledge only four studies investigating the role of oncogenic markers in laryngeal BSCC have been published [21,22,34,35]. In our study the prognostic value of Ki67/Mib-1, p53 and p27^kip1 ^proteins immunoexpression was analyzed in 16 cases of BSCC of the larynx.

Ki67/Mib-1 is a proliferation marker highly expressed in the majority of tumours [47-49]. The Ki67/Mib-1 proliferation index, however, does not allow an unequivocal discrimination between cancers with different "biological" behaviour. Moreover, the data on the relationship between p53 and Ki67 expression, obtained in different tumour types and from different series are somehow controversial.

p53 and p27^kip1 ^play a pivotal role in regulating the cell cycle. p53 is a nuclear phosphoprotein inhibiting cell cycle progression and inducing apoptosis, both regulated by several target genes. It ultimately functions as a tumour suppressor gene, which can induce, in response to mitogenic stimuli, an arrest of the G1-S transition. p53 mutations are extremely frequent in human cancers, and missense p53 mutations with consequent over-expression of a dysfunctional but otherwise intact protein have been described [46]. In head and neck cancer, data from literature show conflicting evidence concerning the correlation between p53 expression and the most common clinicopathological parameters [36,45,49-51]. Recently p53 expression, together with Cyclin D1 over-expression, has been associated with reduced survival in laryngeal SCC [25].

p27^kip1 ^plays an important role in protecting cells from an exuberant proliferation, by regulating cyclin dependent kinase activity thus inhibiting the G1-S transition [52]. Its function results inactive in several human tumours, either by degradation via the SCF-type E3 ubiquitin ligase, Skp2 [53] or by sequestration through Cyclin D3 [54]. In the head and neck cancer, p27^kip1 ^expression has been considered as an independent prognostic factor, able to correlate low-expressing tumours with unfavourable course. Jordan et al. [55] demonstrated that p27^kip1 ^was significantly reduced in oral dysplasia and carcinomas, while it was retained in normal epithelial controls. Interestingly, p27^kip1 ^levels displayed a significant reduction in high grade in comparison with low grade dysplasia, suggesting that changes in p27^kip1 ^expression may be an early event in oral carcinogenesis. In the larynx, a reduced expression of p27^kip1^protein correlates with progression in precancerous lesions [26] and with an aggressive biological behaviour in laryngeal SCC [30]. According to Pruneri et al [27] and Pignataro et al [33], immunohistochemical evaluation of p27^kip1 ^expression is a significant independent predictor of prognosis in laryngeal carcinoma, as previously stated by Fan et al. [28] who found p27^kip1 ^expression to be the most powerful prognostic factor among gender, tumour size, lymph node metastasis, stage of disease and p53 expression. Up to now, the role of p27^kip1 ^as prognosticator has never been investigated in BSCC of the larynx.

In our series, p27^kip1 ^immunohistochemical levels were nearly abolished in all DOD patients, and highly expressed in a large fraction of the patients with NED. p53 and Ki67 were globally highly expressed both in the patients with NED and in the DOD group, with a slightly lower expression in the former samples compared to the DOD patients. The correlation between p27^kip1 ^expression and p53 or to Ki67/Mib-1 expression in DOD and NED patients groups was not statistically significant although there was a trend suggesting that it was inverse. The finding that both KI67 and p53 are over-expressed in a relevant fraction of our patients exhibiting poor outcome is in agreement with numerous published studies attributing to the above proteins a role as predictors of adverse prognosis in several human tumour types, including head and neck cancers. Never less, Ki67 and p53 expression were widely and abundantly retained also in patients in NED status, and were not able to discriminate, in a significant manner, between different outcomes. The result is not surprising in itself, considering the high proliferative capability and potential of aggressiveness of BSCC when compared to different histotypes.

Another interesting consideration comes out from the observation that p27^kip1 ^expression is mainly localized in the focal areas of squamous cell carcinoma, supporting its role in cell differentiation. We did not find any association between squamous component representation and prognosis. In addition p27 expression, as well as the expression of Ki67 and p53, was exclusively evaluated in the pure basaloid areas.

The basaloid nests showed a p27^kip1 ^positivity that, when present, was specifically polarized and located in the cells toward the central necrosis, some of which with a squamous-like appearance, whilst the cells at the invading front of the tumour, showing peripheral nuclear palisading arrangements, were negative for p27^kip1 ^and intensely positive for p53, and Ki67/Mib-1.

To conclude, this study about cell cycle regulators involvement in BSCC of the larynx seems to confirm published data showing a direct correlation between decreased expression of p27^kip1^and poor prognosis in SCC of the head and neck. Multivariated analysis suggested a clinical prognostic relevance of p27^kip1 ^low expression, which directly correlated with biological aggressiveness and consequent shortened survival. Further studies on larger series of BSCC warrant to better clarify this relationship.

The assessment of p27^kip1 ^expression seems very useful to select patients for more aggressive therapies and to establish which cases can benefit from cell cycle inhibitor suitable drugs candidates [56] (i.e. the epidermal growth factor receptor tyrosine kinase inhibitor ZD1839 [57] and the cyclin-dependent kinase modulator UCN-01 (7-hydroxy-staurosporine) [58] or from new cancer gene therapy [59,60].

## Competing interests

The author(s) declare that they have no competing interests.

## Authors' contributions

GS and DDV equally conceived of the study, participated in its design, coordination and helped to draft the manuscript. SS made substantial contributions to acquisition, analysis and interpretation of data. GM helped to analyse data and has been involved in drafting the article. MM performed immunohistochemical study and contributed to analyse data. GQ helped to analyse data and performed multivariate analysis. VG contributed to revise the article. GDR participated in the design of the study and revised the article critically. LI participated in the design of the study, performed immunohistochemical study and helped to analyse data and to draft the manuscript.

All authors read and approved the final manuscript.

## Pre-publication history

The pre-publication history for this paper can be accessed here:



## Figures and Tables

**Table 1:** Follow-up of 16 cases of BSCC of the larynx

**Case**	**Age**	**Sex**	**Site of origin**	**Staging**	**Treatment**	**Follow-up**	**Remarks**
1	44	M	SG	T3N1M0	TL, FND, RT	DOD 7 yrs	LR
2	54	M	SG	T3N0M0	SL, FND	DOD 6 yrs	Lung M
3	57	M	SG	T2N0M0	Laser	NED > 9 yrs	NR after 9 m, ND
4	51	M	G	T3N2M0	TL, FND, RT	NED > 8 yrs	
5	55	M	G	T3N1M0	TL, FND, RT	DOD 7 yrs	NR, LR
6	64	M	SG	T4N0M0	SL, FND, RT	DOD 4 yrs	NR, LR
7	63	M	SG	T2N0M0	SL, FND	NED > 7 yrs	
8	46	M	SG	T3N1M0	TL, FND, RT	AWD >7 yrs	NR after 3 yrs
9	51	M	SG	T3N1M0	TL, FND, RT	DOD 2 yrs	LR
10	67	M	SG	T2N1M0	SL, FND, RT	DOD 5 yrs	LR
11	63	M	SG	T3N1M0	TL, FND, RT	DOD 4 yrs	NR, LR
12	51	M	SG	T4N2M0	SL, FND, RT	DOD 3m	Lung an Liver M
13	69	M	G	T1N0M0	Laser	NED > 6 yrs	
14	64	M	SG	T4N2M0	TL, FND, RT	NED > 6 yrs	
15	54	F	SG	T4N2M0	TL, FND, RT	DOD 4 yrs	NR, Lung M
16	69	M	SG	T3N0M0	TL, FND	DOD > 2 yrs	NR, LR

SG = Supraglottic, G = Glottic, SL = supraglottic laryngectomy; FND = functional neck dissection; RT = radiotherapy; TL = total laryngectomy; DOD = dead of disease; NED = no evidence of disease; AWD = alive with disease; NR = neck recurrence; LR = local recurrence; M = distant metastases; SNS = salvage neck surgery.

**Table 2:** p27^kip1^, p53 and Ki67/Mib-1 antibodies range and median % score

**Case**	**Field 1**	**Field 2**	**Field 3**	**Field 4**	**Field 5**	**Median % score and**
1	10*	15*	10*	13*	15*	13* (10/15)
	20°	20°	18°	16°	16°	18° (16/20)
	30#	30#	32#	28#	30#	30# (28/32)
2	20*	18	20*	18*	20*	20* (18/20)
	31°	30°	27°	28°	30°	30°/27/31)
	28#	26#	24#	28#	30#	28# (24/30)
3	15*	18*	18*	17*	20*	18* (15/20)
	25°	30°	28°	27°	29°	28° (25/30)
	28#	26#	24#	28#	30#	28# (24/30)
4	50*	52*	54*	60*	55*	54* (50/60)
	15°	17°	16°	14°	17°	16° (14/17)
	25#	20#	22#	24#	25#	24# (20/25)
5	5*	7*	7*	5*	4*	5* (4/7)
	25°	28°	27°	30°	25°	27° (25/30)
	30#	31#	35#	28#	30#	30# (28–35)
6	16*	20*	18*	15*	16*	16* (15/20)
	18°	12°	12°	13°	14°	13° (13/18)
	25#	20#	22#	24#	25#	24# (20/25)
7	15*	14*	15*	20*	18*	15* (14/20)
	5°	4°	4°	5°	3°	4° (3–5)
	20#	21#	22#	20#	25#	21# (20/25)
8	52*	54*	55*	58*	60*	55* (52/60)
	2°	2°	4°	3°	3°	3° (2/3)
	30#	29#	32#	20#	31#	30# (20/31)
9	4*	6*	7*	8*	8*	7* (4/8)
	7°	6°	5°	8°	6°	6° (5/8)
	38#	34#	33#	30#	37#	34# (30/38)
10	20*	18*	20*	22*	20*	20* (18/22)
	12°	14°	18°	10°	20°	14° (10/20)
	30#	32#	28#	28#	35#	30# (28/35)
11	15*	17*	16*	18*	20*	17* (15/20)
	20°	21°	20°	18°	24°	20° (20/24)
	28#	35#	30#	35#	30#	30# (28/35)
12	20*	22*	20*	18*	20*	20* (18/22)
	27°	26°	24°	24°	27°	26° (24/27)
	30#	30#	26#	26#	28#	28# (26/30)
13	54*	50*	58*	54*	50*	54* (50/58)
	5°	5°	4°	7°	6°	5° (4/7)
	8#	7#	7#	5#	8#	7# (5/8)
14	4*	6*	8*	6*	8*	6* (4/8)
	15°	15°	12°	13°	14°	14° (12/15)
	25#	24#	22#	25#	24#	24# (22/25)
15	10*	10*	12*	18*	12*	12* (10/18)
	23°	22°	20°	22°	20°	22° (20/23)
	35	34#	34#	30#	34#	34# (30/35)
16	15*	17*	18*	16*	15*	16* (15/18)
	30°	28°	30°	32°	28°	30° (28/32)
	40#	38#	35#	35#	30#	35# (30/40)

(*) p27^kip1 ^(°) p53 (#) ki/67 Mib-1

**Table 3:** Immunohistochemical expression of p27^kip1^, p53 and Ki67/Mib-1

Case	p27^kip1^	p53	Ki67	Outcome
**1**	**0**	**1**	**1**	**DOD**
**2**	**0**	**1**	**1**	**DOD**
**3**	**0**	**1**	**1**	**NED**
**4**	**1**	**1**	**1**	**NED**
**5**	**0**	**1**	**1**	**DOD**
**6**	**0**	**1**	**1**	**DOD**
**7**	**0**	**0**	**1**	**NED**
**8**	**1**	**0**	**1**	**AWD**
**9**	**0**	**0**	**1**	**DOD**
**10**	**0**	**1**	**1**	**DOD**
**11**	**0**	**1**	**1**	**DOD**
**12**	**0**	**1**	**1**	**DOD**
**13**	**1**	**0**	**0**	**NED**
**14**	**0**	**1**	**1**	**NED**
**15**	**0**	**1**	**1**	**DOD**
**16**	**0**	**1**	**1**	**DOD**

(*) p27^kip1 ^(°) p53 (#) ki/67 Mib-1

**Table 4:** Multivariate analysis

Step		Tolerance	Min. Tolerance	F to Enter	Wilks' Lambda
0	age	1.000	1.000	.070	.995
	size	1.000	1.000	2.827	.832
	n	1.000	1.000	.583	.960
	radio	1.000	1.000	.583	.960
	p27	1.000	1.000	8.750	.615
	ki67	1.000	1.000	1.750	.889
	p53	1.000	1.000	3.500	.800

1	age	.944	.944	.561	.590
	size	.982	.982	.955	.573
	n	.954	.954	1.175	.564
	radio	.954	.954	1.175	.564
	ki67	.800	.800	.000	.615
	p53	.931	.931	.731	.583

Test of Function(s)	Wilks' Lambda	Chi-square	df	Sig.	

1	.615	6.554	1	.010	

**Table 5:** Multivariate analysis

**Variables Entered/Removed**^a, b, c, d^									
		Wilks' Lambda							
		
						Exact F			
						
Step	Entered	Statistic	df1	df2	df3	Statistic	df1	df2	Sig.

1	p27	.615	1	1	14.000	8.750	1	14.000	.010

At each step, the variable that minimizes the overall Wilks' Lambda is entered.

a. Maximum number of steps is 16.

b. Minimum partial F to enter is 3.84.

c. Maximum partial F to remove is 2.71.

d. F level, tolerance, or VIN insufficient for further computation.
